# Inferring disease severity in rheumatoid arthritis using predictive modeling in administrative claims databases

**DOI:** 10.1371/journal.pone.0226255

**Published:** 2019-12-18

**Authors:** Urmila Chandran, Jenna Reps, Paul E. Stang, Patrick B. Ryan

**Affiliations:** Janssen Research and Development, Titusville, New Jersey, United States of America; Campus Bio-Medico University of Roma, ITALY

## Abstract

**Background:**

Confounding by disease severity is an issue in pharmacoepidemiology studies of rheumatoid arthritis (RA), due to channeling of sicker patients to certain therapies. To address the issue of limited clinical data for confounder adjustment, a patient-level prediction model to differentiate between patients prescribed and not prescribed advanced therapies was developed as a surrogate for disease severity, using all available data from a US claims database.

**Methods:**

Data from adult RA patients were used to build regularized logistic regression models to predict current and future disease severity using a biologic or tofacitinib prescription claim as a surrogate for moderate-to-severe disease. Model discrimination was assessed using the area under the receiver (AUC) operating characteristic curve, tested and trained in Optum Clinformatics^®^ Extended DataMart (Optum) and additionally validated in three external IBM MarketScan^®^ databases. The model was further validated in the Optum database across a range of patient cohorts.

**Results:**

In the Optum database (n = 68,608), the AUC for discriminating RA patients with a prescription claim for a biologic or tofacitinib versus those without in the 90 days following index diagnosis was 0.80. Model AUCs were 0.77 in IBM CCAE (n = 75,579) and IBM MDCD (n = 7,537) and 0.75 in IBM MDCR (n = 36,090). There was little change in the prediction model assessing discrimination 730 days following index diagnosis (prediction model AUC in Optum was 0.79).

**Conclusions:**

A prediction model demonstrated good discrimination across multiple claims databases to identify RA patients with a prescription claim for advanced therapies during different time-at-risk periods as proxy for current and future moderate-to-severe disease. This work provides a robust model-derived risk score that can be used as a potential covariate and proxy measure to adjust for confounding by severity in multivariable models in the RA population. An R package to develop the prediction model and risk score are available in an open source platform for researchers.

## Introduction

Insurance claims databases are being increasingly employed in drug safety studies, due to the advantages of large sample size, representativeness of patients in routine practice, comprehensive capture of all health encounters, and relative efficiency compared with randomized clinical trials and patient registers. However, confounding by indication has been viewed as a major challenge for observational database studies of rheumatic diseases due to the strong relationship between disease activity and treatment choice [1]. Since health insurance claims databases collect data mainly for reimbursement purposes, they lack detailed clinical data considered critical for conditions. For instance, rheumatoid arthritis (RA) disease activity, which is one of the most frequently used factors indicating poor prognosis is generally assessed by the number of swollen and tender joint counts, serum levels of C-reactive protein and erythrocyte sedimentation rate, and physical and functional disability [2]. However, such clinical and laboratory data to assess disease activity are not routinely or explicitly captured in an administrative claims database, which limit ability of researchers to minimize imbalances due to confounding by disease severity when comparing different treatments in RA patients using large claims databases. Hence, there is a methological gap to identify reproducible scientific methods that allow RA researchers to leverage the power of large administrative claims databases in answering research questions of interest, while handling traditional limitations of incomplete clinical data in these databases.

In the past, claims-based studies in RA have used combinations of drugs, physician visits, joint surgery, and hospital visits in their attempt to adjust for disease severity in their analyses [3–6]. However, there is no model for disease severity that is consistently supported or used in studies of RA conducted with claims data. Recent innovations in statistical computing, such as large-scale regularized regression [7], have enabled data-driven approaches to model fitting, whereby thousands of candidate covariates could be considered when estimating a propensity score, a common statistical confounding adjustment strategy. These techniques have raised the possibility that patient attributes that are not directly observed could be effectively modeled using large sets of observable variables, which are likely correlated with the variable of interest, and a model-based value alone could be used as a proxy for the relevant clinical variable(s).

The American College of Rheumatology (ACR) [8] and the European League Against Rheumatism (EULAR) guidelines [9] recommend advanced therapies including tumor necrosis factor inhibitors (TNFi) biologics, non-TNFi biologics (such as interleukin-6, IL-6 inhibitors), or a Janus kinase inhibitor (JAKi), tofacitinib, all with or without a traditional DMARD, if disease activity continues to persists despite treatment with conventional or traditional disease modifying anti-rheumatic drugs (DMARDs). Dispensing of prescribed therapies is observable in claims databases.

Therefore, in this study, using biologic or JAKi dispensing as a surrogate for disease severity, we sought to develop a prediction model framework [10] that could discriminate between RA patients who were prescribed advanced therapies (biologic or JAKi) and RA patients who were not prescribed a biologic or JAKi in a specified time-period. Since these drugs are only approved for use in patients with moderate-to-severe disease, we can infer with high specificity that patients with these drugs are moderate-to-severe. Conversely, patients who do not receive these treatments are assumed to be not yet moderate-to-severe, recognizing this assumption may have some measurement error amongst patients who are inadequately controlled on DMARDs but unwilling or unable to receive biologic treatment. In this regard, while the prediction problem we proposed to train is: ‘Amongst patients with active RA, which patients are exposed to biologics?’ and we are limited to using the available medical history with associated conditions, drugs, procedures and health service utilization markers present in claims data, our desired application of the resulting model is to estimate, ‘Amongst patients with active RA, which patients have moderate-to-severe disease?’ and use that estimate as a proxy for disease severity in subsequent applications of claims analyses. The aim of this study is therefore to propose a methodological alternative to minimizing imbalances due to disease severity when conducting research in data sources that have limited clinical and lab data.

## Materials and methods

### Data sources

A model to predict RA disease severity based on prescription of a biologic or JAKi was developed using deidentified data in the US-based administrative claims database, Optum De-Identified Clinformatics^®^ Extended Data Mart Database-Socio-Economic Status (hereafter referred to as “Optum”) and externally validated in other US administrative claims databases, IBM MarketScan^®^ Commercial Claims & Encounters Database (referred to as “IBM CCAE”), IBM MarketScan^®^ Medicare Supplemental Database (referred to as “IBM MDCR”), and IBM MarketScan^®^ Multi-State Medicaid Database (referred to as “IBM MDCD”). All databases were standardized into the Observational Medical Outcomes Partnership (OMOP) Common Data Model (CDM) [11,12]. A brief description of these databases is provided below:

The Optum De-Identified Clinformatics^®^ Extended Data Mart database (Optum, Eden Prairie, MN) is an adjudicated administrative health claims database for members with private health insurance, who are fully insured in commercial plans or in administrative services only, Legacy Medicare Choice Lives (prior to January 2006), and Medicare Advantage (Medicare Advantage Prescription Drug coverage starting January 2006). IBM CCAE represent data from individuals enrolled in US employer-sponsored insurance health plans. The data include adjudicated health insurance claims as well as enrollment data from large employers and health plans who provide private healthcare coverage to employees, their spouses, and dependents. IBM MDCR represents health services of retirees in the United States with primary or Medicare supplemental coverage through privately insured fee-for-service, point-of-service, or capitated health plans. IBM MDCD includes adjudicated US health insurance claims for Medicaid enrollees from multiple states and includes hospital discharge diagnoses, outpatient diagnoses and procedures, and outpatient pharmacy claims.

The New England Institutional Review Board determined that studies conducted in Optum, IBM CCAE, IBM MDCR, and IBM MDCD are exempt from study-specific IRB review, as these studies do not qualify as human subjects research. All patient data included in this study were deidentified.

### Study population

The study population was defined as patients with a diagnosis claim for RA (index date) during the study period, Jan 1, 2001-Dec 31, 2016 that met the following criteria: 18 years or older as on index date; 730 days of continuous observable time prior to the index date; at least one diagnosis claim for RA (using ICD-9-CM diagnosis codes for RA: 714.0, 714.1, 714.2; see S1 Table for full code list) in both the 0–365 days and 366–730 days prior to the index date, and have at least one prescription claim for methotrexate, sulfasalazine, hydroxychloroquine, or leflunomide in the 365 days prior to index. Our RA definition therefore, ensures each patient to have diagnosis codes for RA on at least two separate visits along with a prescription claim for RA therapy, which is consistent with the findings of a systematic review of validated RA patient definitions in administrative claims data which reported the highest positive predictive values (PPVs) when using 2 or more RA diagnosis or procedure codes for RA and included use of prescriptions with an RA indication [13]. Patients with RA who had been prescribed a biologic or JAKi prior to the index date were excluded to minimize enriching the dataset with patients that were probably severe prior to the study start. Patients were required to be observed for the entire time-at-risk (90 days or 730 days), and subjects who did not have sufficient time-at-risk (TAR), independent of whether or not they had the outcome, were excluded from the analysis.

Subjects were also excluded if they had a diagnosis of juvenile idiopathic arthritis, psoriasis, psoriatic arthritis, ankylosing spondylitis, Crohn’s disease, or ulcerative colitis (see S1 Table) any time prior to index date, as some non-biologic and/or biologic DMARDs are indicated for treatment of these conditions. Patients were also excluded if they had procedure codes for intravenous administration of methotrexate during the pre- or post-index period, as these would suggest treatment for cancer.

As the predictive model was developed with the aim to be reproducible, a standardized clinical vocabulary for medical conditions, SNOMED-CT was utilized. Due to the existence of mappings between SNOMED-CT and other vocabularies, medical condition code sets using SNOMED-CT can be readily translated into other vocabularies such as ICD-9-CM or ICD-10-CM. See S1 Table for concept sets used in this paper (available in SNOMED-CT and translated to other vocabularies).

### Outcome of interest

The outcome of interest for this study was the first prescription claim for a TNFi biologic (infliximab, adalimumab, golimumab, certolizumab pegol, etanercept), non-TNFi biologic (abatacept, rituximab, tocilizumab, anakinra), or JAKi (tofacitinib) during the TAR period. We used two TAR definitions: 1) starting 1 day after index through 90 days and 2) starting 1 day after index through 730 days. The TAR period of 90 days was selected as a proxy for current disease severity and TAR period of 730 days for future RA disease severity.

### Candidate covariates

Covariates included in the prediction model were demographics (age, gender, race, ethnicity, index month); conditions (occurrence in past 30 and 365 days); drugs (exposures in past 30 and 365 days), drug ingredient (in past 30 and 365 days), procedures (in past 30 and 365 days), laboratory measurements (in past 30 and 365 days), and risk scores (Charlson, CHADS2, CHADS2VASc, and DCSI). This covariate inclusion was not manually developed, but rather represents all available data in the database. However, a minimum constraint of 20 subjects was required for a covariate to be included in the model.

### Statistical analysis

Patients were classified as having the outcome if there was a prescription of a biologic or tofacitinib during the TAR. The target cohort in Optum was split into test and train datasets, whereby 75% of the data were used to train the model and remaining 25% of the data was used to internally validate the model by comparing the prediction with the observed outcome. The study utilized a prediction model framework that has previously demonstrated reproducibility to facilitate model sharing and offering the ability to perform external validation [10]. Three machine learning algorithms were applied to predict outcome: regularized LASSO (least absolute shrinkage and selection operator) logistic regression, random forest, and gradient boosting machine. The most suitable model for the prediction question was determined using a process known as 10-fold cross validation. The 10-fold cross validation involves a process where the training data are partitioned into 10 independent data sets and for each set, a model is trained using the combination of the remaining 9 datasets and then validated on the left-out set. This is a technique used to calculate a fair estimate of the model performance for various model complexities (hyper-parameter settings), while using the whole training set. For LASSO logistic regression there is only one hyper-parameter that controls complexity (lambda), and the optimal lambda value was determined in this study using an automatic search.

Model discrimination was assessed using the area under the receiver operating characteristic curve (AUC) and calibration was assessed using calibration plots. The AUC corresponds to the probability that a randomly selected person with the outcome is assigned a higher risk of the outcome by the model than a randomly selected person without the outcome. Calibration plots provided a visual to inspect if a model’s predicted risk matched the observed risk. Specifically, the test set target population was split into deciles based on the model’s predicted risk (e.g., if there were 100 patients in the test set, and the model was applied to them to get their predicted risks, 10 groups of 10 patients are created). For each decile, the mean predicted risk was calculated along with the fraction of the patients in the group who had the outcome. If a model is well calibrated, the mean predicted risk should match the observed fraction of patients in the group who have the outcome. For example, if the mean predicted risk in a group of 10 patients is 10%, then if the model is well calibrated, one patient in the group should have the outcome. The trained model was then additionally validated in IBM CCAE, IBM MDCR, and IBM MDCD to evaluate external model performance. Descriptive summaries of characteristics among patients that were prescribed and those that did not get prescribed a biologic or tofacitinib in the 90 days and 730 days since index were also generated.

Analyses were conducted using open-source tools from OHDSI [14], and the full analysis source code to replicate this study, including the models developed for this paper’s analysis are available at https://github.com/OHDSI/StudyProtocolSandbox/tree/master/RASeverity [15].

### Additional validation analysis

In addition to validating the prediction model in three external claims databases, a separate validation exercise was conducted by plotting the risk score generated from the prediction model against first exposure to the various therapies used in RA patients in the Optum database during January 1, 2017 –December 31, 2017. The year 2017 was chosen as it is the most recent full year of available data and as this data was not included in the development of the prediction model. Furthermore, conducting this additional analysis based on first exposure to therapy also allowed assessing the generalizability of the prediction model to patients at different stages of their disease.

## Results

A total of 68,608 RA patients in the Optum database were eligible for the 90 days TAR analysis, of which 3% (n = 1,916) had a biologic or tofacitinib prescription claim in the 90 days following the index diagnosis. A summary of the descriptive characteristics of the RA patient cohorts for the two TAR periods is shown in Table 1, which shows the younger age and fewer comorbidities among patients who were prescribed a biologic or tofacitinib than those that were not.

**Table 1:** Characteristics of RA patients for TAR 90 days and TAR 730 days in Optum database.

	90 days TAR		730 days TAR	
Characteristic	Patients with outcome, % (n = 1,916)	Patients without outcome, % (n = 66,692)	Patients with outcome, % (n = 4593)	Patients without outcome, % (n = 32,535)
**Demographics**				
Median age (years)	54	63	55	65
Female gender	79	76	79	76
**Medical history**				
Chronic obstructive lung disease	7	11	7	11
Depressive disorder	14	14	13	13
Diabetes mellitus	14	19	14	19
Hyperlipidemia	36	46	36	47
Hypertensive disorder	41	54	41	55
Heart disease	18	28	18	28
Venous thrombosis	3	3	3	3
Malignant neoplastic disease	6	11	6	11
**Medication use**				
Antidepressants	33	29	30	27
Beta blocking agents	17	26	18	26
Calcium channel blockers	13	18	13	19
Diuretics	24	32	26	33
Immunosuppressants	91	74	86	73
Opioids	37	33	35	31
**Comorbidity Indices**				
Charlson comorbidity index, median	2	2	2	2
CHADS2Vasc, median	2	2	2	3
Diabetes Complications Severity Index, median	0	1	0	1

### TAR 90 days prediction models

The 90 days TAR period was used to evaluate performance of the model to predict current disease severity using a prescription claim of biologic or tofacitinib as a surrogate for disease severity. The regularized logistic regression model demonstrated good discrimination in predicting RA patients who were prescribed the therapy versus those that did not, as shown by AUCs of 0.80 and 0.85, in the test and train sets in Optum, respectively (Table 2). The gradient boosting machine and random forest models performed similarly with AUCs of 0.80 and 0.79, respectively. Furthermore, the prediction model trained on Optum also appeared to perform well in additional databases such as IBM CCAE, IBM MDCR, and IBM MDCD, as demonstrated by AUCs of 0.77, 0.75, and 0.77, respectively. Covariates that informed the 90-day TAR model included health service utilization (such as radiologic exams, laboratory tests, office visits, etc.), demographics, prescription claims for immunosuppressants, steroids, DMARDs, pain medications, and other comorbid conditions. For example, covariates that were more frequently observed among patients with a prescription claim for biologic or JAKi included younger age group (45–49 years), prescriptions for immunosuppressants, leflunomide, methotrexate, prednisone, diagnosis of multiple joint pain, procedures for radiologic exam and office visit involving comprehensive medical exam or complex medical decision-making to name a few. Further details on covariates that predicted prescription of a biologic or JAKi can be explored through the interactive online shiny app available at http://data.ohdsi.org/RASeverity/. It is important to note, however that prediction models are not causal models, hence covariates that are predictive of an outcome are not necessarily risk factors for the outcome.

**Table 2:** Logistic regression model AUCs for TAR 90 days and TAR 730 days.

	TAR 90 days			TAR 730 days		
**Test Database**	**AUC**	**Outcome Count**	**Population size**	**AUC**	**Outcome Count**	**Population size**
Optum (Test Model)	0.80 (0.78–0.83)	479	17152	0.78 (0.76–0.79)	1148	9282
Optum (Train Model)	0.85 (0.84–0.86)	1437	51456	0.79 (0.78–0.80)	3445	27846
**Validation databases**	**AUC**	**Outcome Count**	**Population size**	**AUC**	**Outcome Count**	**Population size**
IBM CCAE	0.77 (0.76–0.78)	3202	75579	0.71 (0.70–0.71)	7107	38041
IBM MDCR	0.75 (0.73–0.77)	684	36090	0.71 (0.69–0.72)	1976	21413
IBM MDCD	0.77 (0.74–0.80)	265	7537	0.71 (0.69–0.73)	481	3807

**Fig 1:**
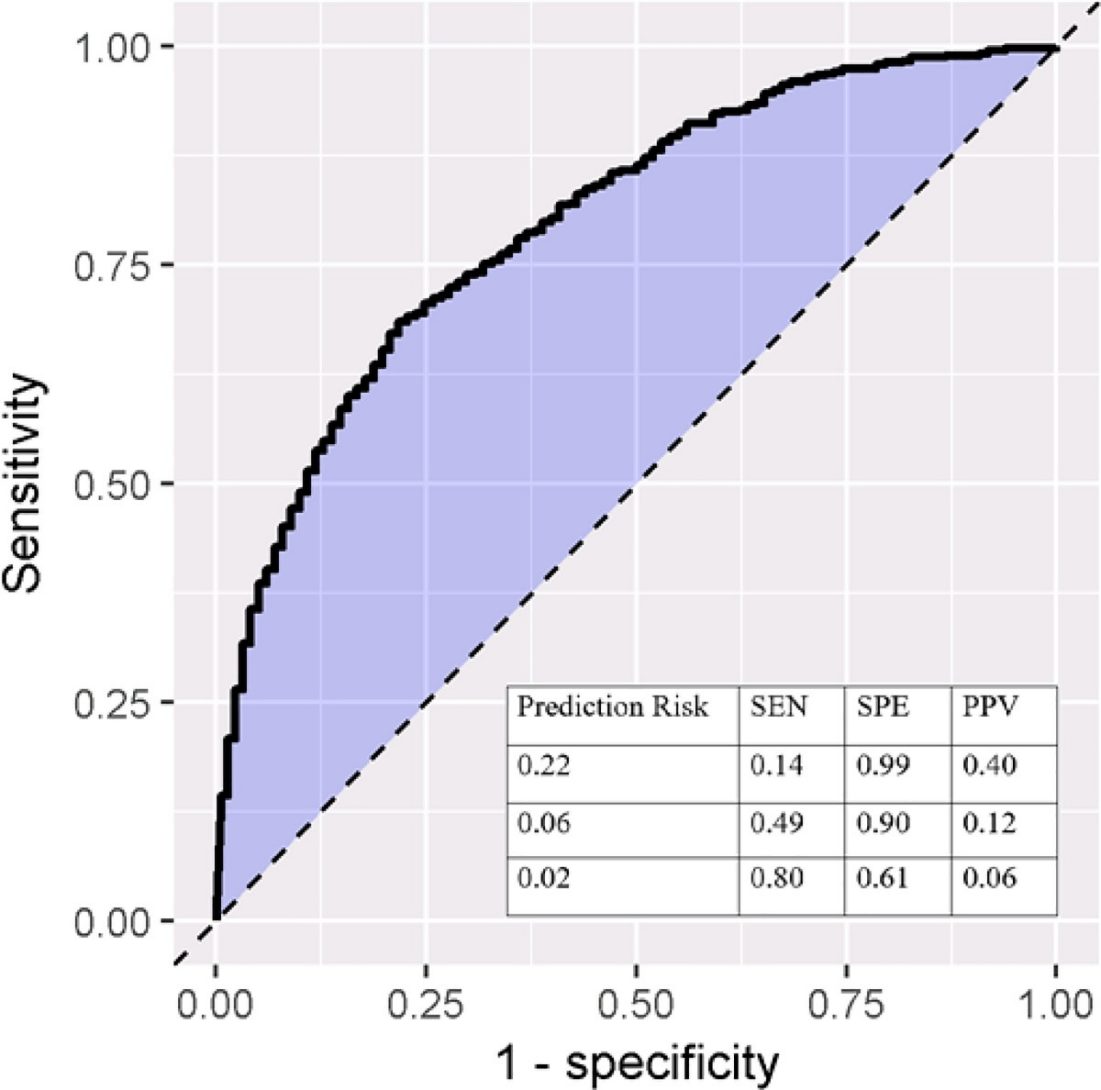
ROC plot for 90 days TAR in Optum. SEN: sensitivity, SPE: specificity, PPV: positive predictive value.

**Fig 2:**
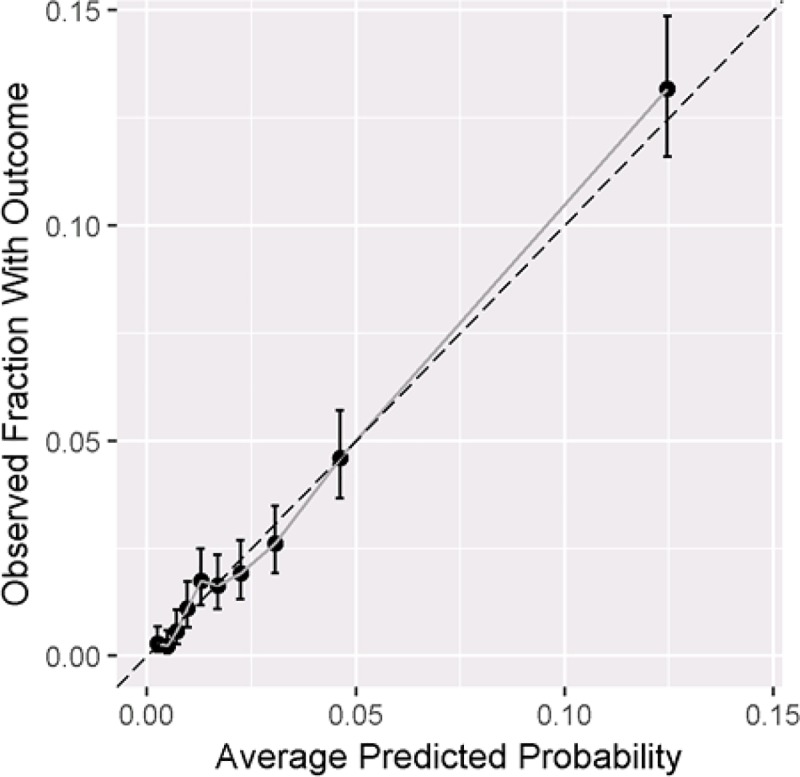
Calibration plot for 90 days TAR in Optum.

The AUC and calibration plots for the Optum model, predicting outcome during the 90 days since index are shown in Figs 1 and 2. The sensitivity, specificity, and positive predictive value for different prediction risk probabilities are provided in Fig 1. As an illustration, if the prediction risk is 0.22, whereby patients assigned a risk of 0.22 or more by the model would be classified as moderate-to-severe RA and those assigned a risk of less than 0.22 being classified as non-severe RA, we would correctly find 14% of patients with a biologic being classified as having moderate-to-severe RA, while incurring a 1% false positive rate (1-specificity) and a positive predictive value of 40%. This corresponds to the fact that approximately 4 in 10 patients that we classify as having moderate-to-severe disease will have a biologic or JAKi in the next 90 days. Reducing the prediction threshold improves the proportion of patients with a biologic or JAKi claim being correctly classified as having moderate-to-severe disease by the model (i.e., increased sensitivity). The calibration plot in Fig 2 shows that the observed fraction of patients in each decile and the corresponding mean predicted risk for each decile (i.e., the ten dots) fall along the x = y line, indicating excellent model calibration.

### TAR 730 days prediction models

To evaluate model performance for a longer follow-up period, prediction models were regenerated using 730 days as TAR period since index. A total of 37,128 patients with RA were identified in the Optum database, and as expected, a higher proportion of patients (12.4%, n = 4,593) had a prescription claim for a TNFi- or non-TNFi biologic, or tofacitinib in the 730 days following the index date, compared with the 90-day results. Covariates included in the 730-day TAR model were similar to those that informed the 90-day TAR model.

The AUCs for the Optum model trained using regularized logistic regression for predicting prescription of a biologic or tofacitinib in the 730 days since index, as well as AUCs from the three additional databases (utilized for external validation) are presented in Table 2. The model demonstrated good discrimination in predicting RA patients who were prescribed the therapy versus those that did not as shown by AUCs of 0.78 and 0.79, on the test and train set, respectively. The gradient boosting machine and random forest models showed similar performance to the logistic regression model with AUCs of 0.77 and 0.75, respectively. Notably, the prediction model developed on Optum also appeared to perform well in external validation databases, as demonstrated by AUC of 0.71 across IBM CCAE, IBM MDCR, and IBM MDCD.

**Fig 3:**
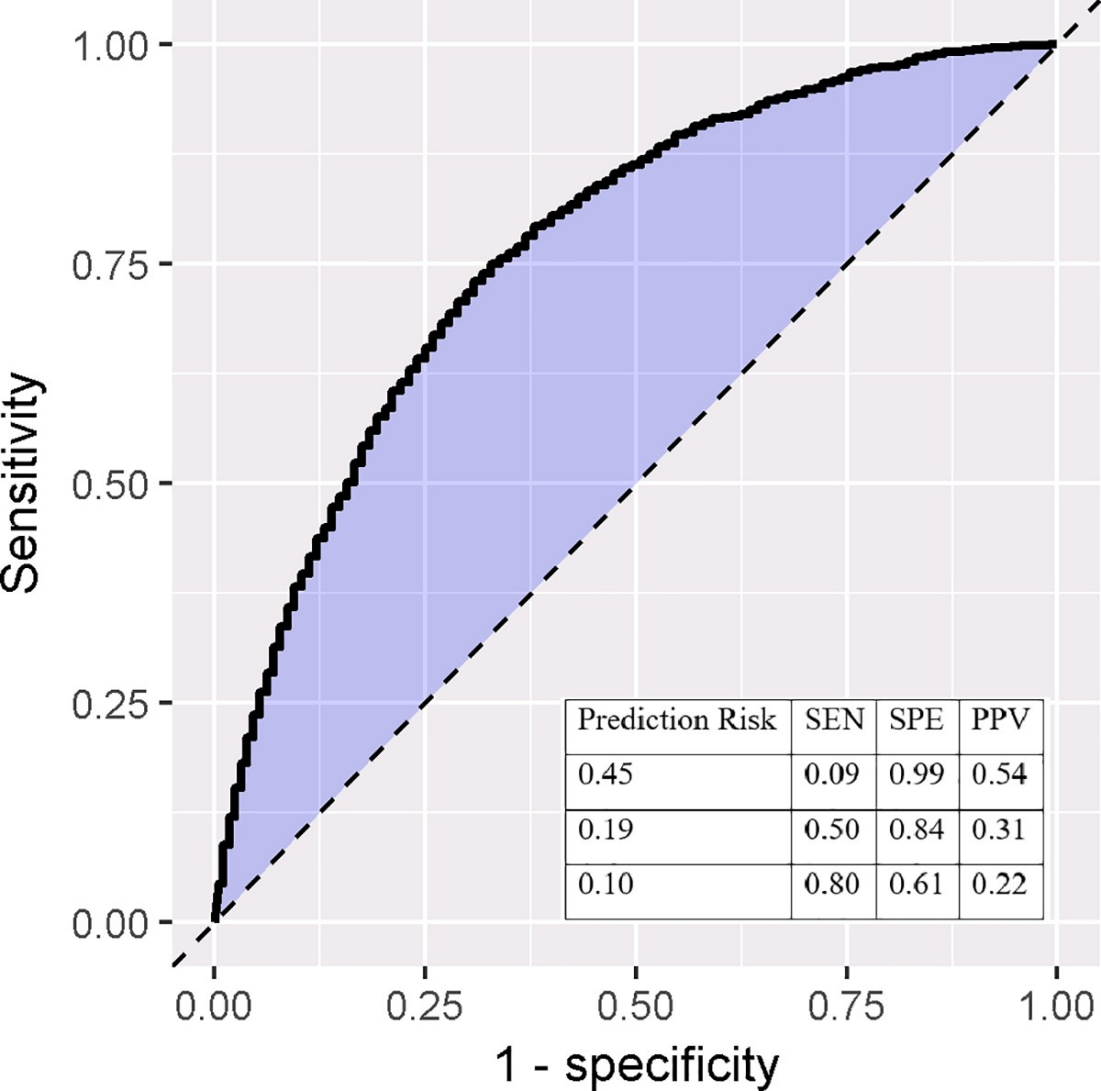
ROC plot for 730 days TAR in Optum. SEN: sensitivity, SPE: specificity, PPV: positive predictive value.

**Fig 4:**
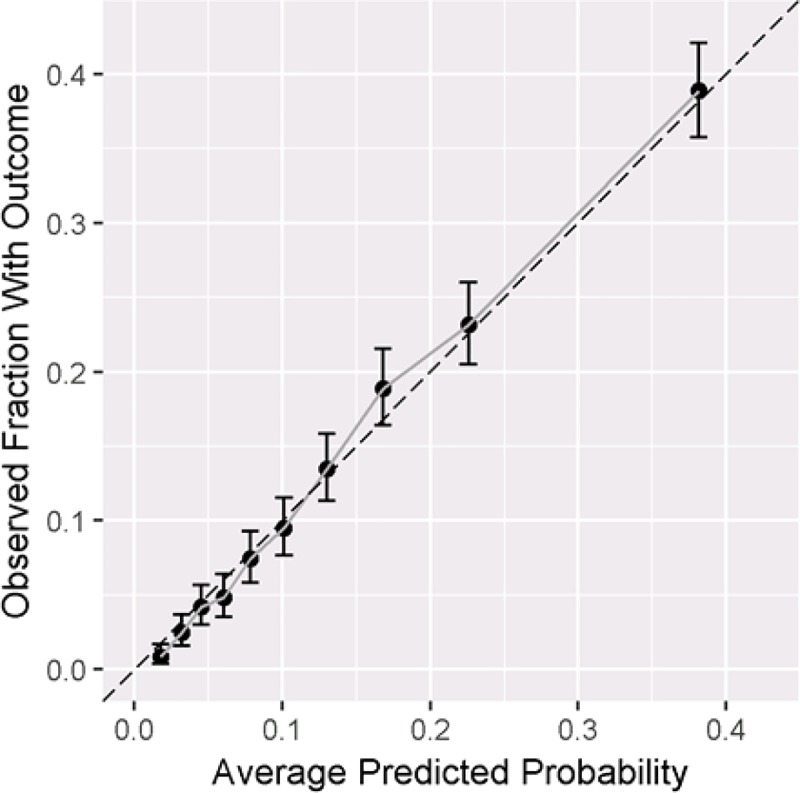
Calibration plot for 730 days TAR in Optum.

The AUC and calibration plots for the Optum model, predicting outcome during the 730 days since index are shown in Figs 3 and 4. With a longer TAR period, a prediction probability of 0.19 correctly identified 50% of patients with a biologic or tofacitinib being classified as having moderate-to-severe RA, while only incurring a 16% false positive rate. With these parameters, close to one-third of all patients that we classify as having moderate-to-severe disease will have a biologic claim. The calibration plot (Fig 4) shows that the model was well calibrated as the results fall along the x = y line, indicating the model’s predicted risks match the observed risks.

### Additional validation results

**Fig 5:**
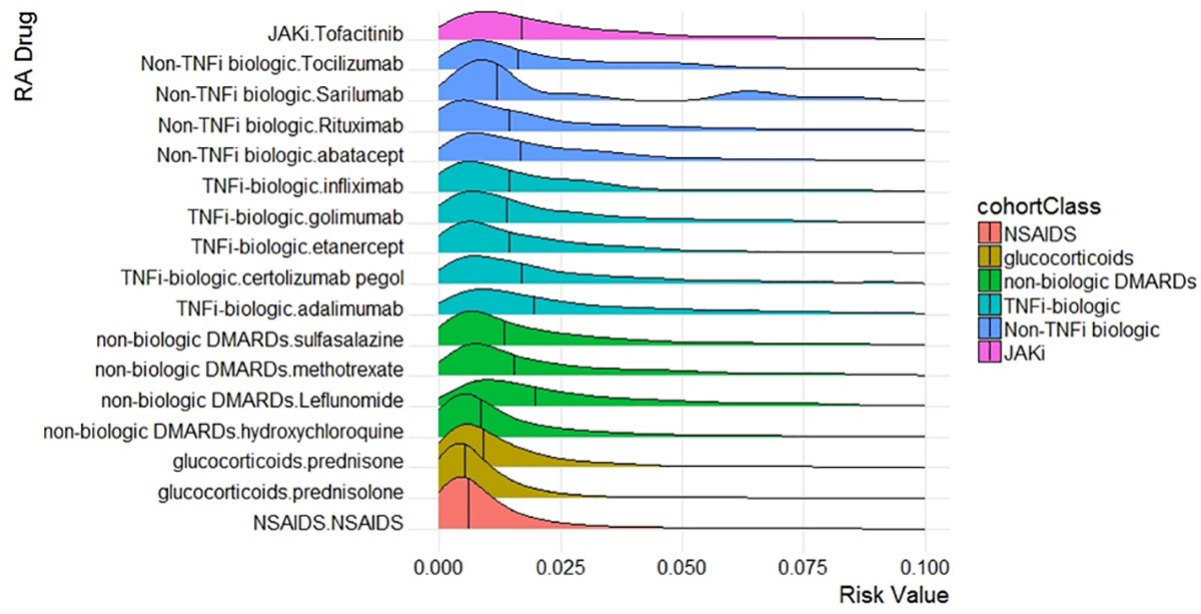
Ridgeline plot of risk score from prediction model and RA therapy.

The ridgeline plot (Fig 5) shows the proxy severity risk score distributions at the time point of an RA drug being dispensed across drugs used to treat RA. This demonstrates the extent to which the risk scores generated by the prediction model differentiated RA patients exposed to different drugs and classes of therapies. The plot shows that the median risk score for NSAIDs, corticosteroids, and some of the non-biologic DMARDs are lower than for the TNFi- and non-TNFi biologic classes and JAKi therapies, suggesting that the prediction model assigned lower scores to earlier lines of therapy than more advanced therapies. The low absolute risk scores overall reflect the small proportion of RA patients that were prescribed advanced therapies in the database.

### Prediction model application

The developed model provides a risk probability between 0 and 1 of being prescribed a biologic or tofacitinib therapy during the TAR relative to a given date for a given patient. This risk score for each patient can be included as a covariate in a multivariable regression model as a correlate of RA disease severity. The full analysis source code to replicate this study, including the models developed for this paper’s analysis are available at https://github.com/OHDSI/StudyProtocolSandbox/tree/master/RASeverity [15]: This site also includes instructions to apply the models to calculate the risk probabilities for a set of patients and corresponding dates in any database mapped to the OMOP CDM. In addition, an interactive online shiny app for exploring the models generated as part of this study’s analysis and their performance data can be found in an open source platform at http://data.ohdsi.org/RASeverity/.

## Discussion

In this paper, we evaluated if RA patients requiring treatment with a TNFi- or non-TNFi biologic or tofacitinib, can be discriminated from patients that were not prescribed these drugs during a short-term and long-term period in US administrative claims databases. Results of prediction models developed and internally validated in the Optum database and externally validated in IBM CCAE, IBM MDCR, and IBM MDCD databases showed good model performance both while predicting proxy disease severity in the short term (90 days), as well as in the future (730 days) since index diagnosis. Model performance was similar across multiple statistical approaches, and excellent calibration ability was demonstrated by calibration plots. In an additional validation exercise, the model also appeared to assign different risk scores to RA patients receiving more and less advanced therapies and demonstrated generalizability across a range of RA patients at various lines of therapy. This research attempts to minimize clinical data limitations of health insurance claims databases by developing a surrogate disease severity score that can be potentially used to adjust for RA severity in pharmacoepidemiologic studies and widely applied to other databases utilizing a common data structure.

The baseline characteristics of RA patients included in the present analyses showed that patients that received a prescription claim for biologics or tofacitinib either in the short or long term were younger than patients that did not receive these therapies in the same time periods. This observation is consistent with past findings [16]. In published literature, studies evaluating claims-based indices for RA severity have observed low correlation between these indices and disease activity indices traditionally used in clinical practice [17–19]. Only use of treatment variables was also concluded to be an unsatisfactory approach to classify patients according to severity in another study [20]. A recently published methods study suggested that data-based deep learning models that utilized a combination of variables predicted future RA disease activity scores more accurately than a clinical disease activity index [21].

In our analysis, we used large-scale analytics that included several thousand variables from the entire claims database to predict which patient received biologic or tofacitinib. The use of prescription claims for advanced therapies as proxy for disease activity and severity represents one approach to development of a risk score, and there may be others. Our main aim was to develop a prediction model that could differentiate between RA patients who get prescribed advanced therapies at a future date, as one method to facilitate scientific research using claims data that have limited clinical information. Hence, the model was informed by regulatory approved indication and RA treatment guidelines, which recommend the use of TNFi-biologics, non-TNFi biologics, or JAKi (tofacitinib) in moderate or severe RA disease activity [8, 9], as a key underlying assumption regarding the true severity status.

Although this study was performed as an effort to fill a gap in use of administrative claims databases to conduct scientifically rigorous analyses, the comparable performance of both 90-day and 730-day TAR models also suggests clinical implications. The model to predict prescription for advanced therapies up to 2 years forward performed as well as the model that predicted prescription of advanced therapy in 90 days. This finding suggests that identifying patients eligible to receive advanced therapies in the short term could also inform the same in the future. The strong correlation (Pearson r = 0.81 on test set patients) between the 90 days and 730 days prediction models further demonstrates that while each model may be built slightly differently, there are also several factors common to both models.

Both current and future RA proxy severity prediction models performed well when applied to three additional databases, thus lending reassurance in applicability of the model to databases with differing population. However, this does not guarantee similar performance in other databases that may reflect different patient population characteristics. Nevertheless, to investigate the generalizability of the model, we assessed the model performance using data on a range of cohorts of RA patients that did not inform the model. The results demonstrated that the model was able to differentiate between patients prescribed earlier lines of therapy (these patients had lower risk scores for the proxy RA severity variable) from those prescribed advanced therapies like biologics and JAKi. This validation exercise provided reassurance in the model’s discriminating ability as well as generalizability to RA patients at different stages of their disease. Future research would involve validation of our model against alternative disease severity indices, such as RARBIS [18] or CIRAS [19], which were developed in different populations and data sources.

Limitations of the developed model include its dependence on recorded variables captured in the database, as it is possible that lack of well recorded measurements may have affected model discrimination. Similarly, although use of biologic or tofacitinib usually indicates moderate-to-severe disease, there may be other markers for RA severity that could affect model performance. However, the use of all large sets of observed variables in building the prediction model could potentially minimize some of the confounding due to likely correlation with unobserved characteristics.

Strengths include the prediction framework and open source codes that facilitate sharing of models with other researchers in the field to implement and validate in other databases, a process that would be further accelerated if their databases were in the OMOP CDM. Other advantages of using our RA severity model in an estimation study rather than a propensity score matching model are i) some datasets are too small to perform a sufficient propensity model, whereas our model is trained in large data and we have shown it is transportable, and hence can be used when sample size is small and ii) a large-scale propensity model may match on unnecessary variables which can cause limitations.

## Conclusions

This paper presents a data-driven model of an RA severity score that demonstrated good discrimination across multiple claims databases to identify patients with a prescription claim for advanced therapies during different time-at-risk periods. Integrating this score as a baseline variable into multivariable analyses for risk estimation could help minimize confounding when using large administrative claims databases to answer important research questions about RA patient outcomes.

## Supporting information

S1 Table List of SNOMED concept IDs and mapped codes used in the study. (DOCX)
