# Supplementary material for: Inferring disease severity in rheumatoid arthritis using predictive modeling in administrative claims databases
Source: PLoS One. 2019 Dec 18;14(12):e0226255. doi: 10.1371/journal.pone.0226255 (PMC6919633; doi:10.1371/journal.pone.0226255)
Supplement: S1 Table — (DOCX) [file pone.0226255.s001.docx]

**Supporting information**

**S1 Table.** List of SNOMED concept IDs and mapped codes used in the study

| **Diagnoses/Procedures/**  **Drugs** | **Concept IDs and codes** |
| --- | --- |
| Rheumatoid arthritis | ***SNOMED***: 69896004  ***ICD9CM***: 714.0, 714.1, 714.2  ***ICD10CM***:M05,M05.0,M05.01,M05.02,M05.03,M05.04,M05.05,M05.06,M05.07,M05.11,M05.12,M05.13,M05.14,M05.15,M05.16,M05.17,M05.2,M05.21,M05.22,M05.23,M05.24,M05.25,M05.26,M05.27,M05.31,M05.32,M05.33,M05.34,M05.35,M05.36,M05.37,M05.41,M05.42,M05.43,M05.44,M05.45,M05.46,M05.47,M05.51,M05.52,M05.53,M05.54,M05.55,M05.56,M05.57,M05.6,M05.61,M05.62,M05.63,M05.64,M05.65,M05.66,M05.67,M05.7,M05.71,M05.72,M05.73,M05.74,M05.75,M05.76,M05.77,M05.8,M05.81,M05.82,M05.83,M05.84,M05.85,M05.86,M05.87,M06,M06.0,M06.01,M06.02,M06.03,M06.04,M06.05,M06.06,M06.07,M06.8,M06.81,M06.82,M06.83,M06.84,M06.85,M06.86,M06.87,M05.9,M06.9,M05.012,M05.071,M05.252,M05.329,M05.441,M05.462,M05.522,M05.652,M05.769,M05.812,M05.829,M05.859,M06.012,M06.052,M06.079,M05.039,M05.159,M05.249,M05.272,M05.279,M05.351,M05.472,M05.511,M05.539,M05.542,M05.571,M05.752,M06.069,M06.821,M05.059,M05.129,M05.131,M05.231,M05.451,M05.549,M05.70,M05.712,M05.861,M06.851,M06.852,M05.029,M05.041,M05.042,M05.061,M05.072,M05.151,M05.152,M05.359,M05.459,M05.531,M05.561,M05.60,M05.651,M05.721,M05.761,M05.762,M05.869,M05.871,M05.879,M06.80,M06.869,M05.079,M05.162,M05.212,M05.321,M05.322,M05.579,M05.619,M05.671,M05.739,M05.79,M05.80,M05.839,M05.862,M05.89,M06.062,M05.021,M05.319,M05.352,M05.379,M05.429,M05.471,M05.639,M05.819,M06.021,M06.051,M05.022,M05.121,M05.232,M05.311,M05.331,M05.342,M05.362,M05.421,M05.569,M05.729,M05.811,M06.032,M06.822,M06.842,M06.862,M05.019,M05.032,M05.049,M05.139,M05.169,M05.262,M05.312,M05.369,M05.431,M05.461,M05.521,M05.59,M05.659,M05.69,M05.719,M05.759,M06.072,M06.812,M06.832,M06.859,M06.879,M06.88,M05.062,M05.20,M05.261,M05.332,M05.361,M05.371,M05.452,M05.479,M05.642,M05.649,M05.722,M05.751,M05.831,M06.042,M06.09,M06.811,M05.052,M05.069,M05.09,M05.142,M05.149,M05.161,M05.251,M05.39,M05.439,M05.49,M05.612,M05.631,M06.022,M06.029,M06.039,M06.049,M06.059,M06.071,M06.831,M06.839,M06.849,M05.031,M05.119,M05.122,M05.132,M05.172,M05.339,M05.341,M05.432,M05.552,M05.559,M05.562,M05.572,M05.732,M05.749,M05.842,M05.00,M05.111,M05.222,M05.372,M05.841,M05.872,M06.89,M05.051,M05.112,M05.141,M05.229,M05.269,M05.271,M05.469,M05.532,M05.541,M05.551,M05.622,M05.629,M05.641,M05.662,M05.772,M06.019,M06.872,M05.221,M05.241,M05.259,M05.29,M05.411,M05.412,M05.419,M05.442,M05.519,M05.529,M05.611,M05.711,M05.742,M05.771,M05.779,M05.832,M06.00,M06.829,M05.171,M05.211,M05.349,M05.621,M05.632,M05.679,M05.821,M05.822,M05.849,M05.852,M06.061,M06.819,M06.861,M05.011,M05.179,M05.219,M05.239,M05.242,M05.422,M05.449,M05.512,M05.661,M05.669,M05.672,M05.731,M05.741,M05.851,M06.011,M06.031,M06.041,M06.08,M06.841,M06.871 |
| Psoriasis | ***SNOMED***: 9014002  ***ICD9CM***: 696.1, 696.8, 694.3  ***ICD10CM***:L40, L40.5, L40.0, L40.1, L40.3, L40.4, L40.8, L40.9, L40.54, L40.50, L40.52, L40.53,L40.59,L40.51 |
| Psoriatic arthritis | ***SNOMED***: 10037162  ***ICD9CM***: 696  ***ICD10CM***: L40.5,L40.54,L40.50,L40.52,L40.53,L40.59,L40.51 |
| Ankylosing spondylitis | ***SNOMED***: 9631008  ***ICD9CM***: 720  ***ICD10CM***:M45,M45.1,M45.2,M45.5,M45.0,M45.6,M45.4,M45.8,M45.9,M45.3,M45.7 |
| Juvenile idiopathic arthritis | ***SNOMED***: 239796000  ***ICD9CM***: 714.32  ***ICD10CM***:M08,M08.2,M08.21,M08.22,M08.23,M08.24,M08.25,M08.26,M08.27,M08.4,M08.41,M08.42,M08.43,M08.44,M08.45,M08.46,M08.47,M08.3,M08.259,M08.472,M08.219,M08.249,M08.261,M08.271,M08.412,M08.422,M08.432,M08.20,M08.411,M08.429,M08.441,M08.442,M08.431,M08.251,M08.479,M08.28,M08.459,M08.231,M08.241,M08.262,M08.451,M08.461,M08.239,M08.252,M08.272,M08.279,M08.40,M08.462,M08.212,M08.221,M08.452,M08.471,M08.211,M08.269,M08.229,M08.232,M08.29,M08.421,M08.439,M08.469,M08.48,M08.222,M08.242,M08.419,M08.449 |
| Crohn’s disease | ***SNOMED***: 34000006  ***ICD9CM***: 555, 555.1, 555.2, 555.9  ***ICD10CM***:K50,K50.0,K50.1,K50.8,K50.9,K50.00,K50.812,K50.814,K50.913,K50.014,K50.914,K50.811,K50.911,K50.10,K50.111,K50.114,K50.80,K50.011,K50.813,K50.90 |
| Ulcerative colitis | ***SNOMED***: 10009900  ***ICD9CM***: 556, 556.1, 556.3, 556.5, 556.6, 556.8, 556.9  ***ICD10CM***:K51,K51.0,K51.3,K51.5,K51.51,K51.8,K51.9,K51.00,K51.314,K51.014,K51.30,K51.514,K51.311,K51.90,K51.814,K51.914,K51.013,K51.50,K51.80,K51.313,K51.011 |
| Non-biologic DMARDS | ***RxNorm ingredient concept codes***: 9524, 6851, 27169, 5521 |
| Intravenous administration of methotrexate | ***RxNorm concept code***: 1655955 |
| TNFi biologics | ***RxNorm ingredient concept codes***: 191831, 819300, 214555, 709271, 327361 |
| Non-TNFi biologics | ***RxNorm ingredient concept codes***: 612865, 121191, 72435, 614391 |
| JAK inhibitor (tofacitinib) | ***RxNorm ingredient concept code***: 1357536 |
